# Prognostic factors for 30-days mortality in eighty years aged and older critically ill patients: a single center retrospective cohort study

**DOI:** 10.3906/sag-2104-359

**Published:** 2021-09-04

**Authors:** Umut Sabri KASAPOĞLU, Osman KAÇMAZ, Abdullah GÖK, Merih YILDIZ EGLEN, Hasan ŞAYAN, Fatih ÇOLAK

**Affiliations:** 1 Department of Pulmonary and Critical Care Medicine, Malatya Training and Research Hospital, Malatya, Turkey; 2 Department of Anesthesiology and Reanimation, Malatya Training and Research Hospital, Malatya, Turkey

**Keywords:** Older, ICU, frailty, modified NUTRIC score, prognostic factors, mortality

## Abstract

**Background/aim:**

Nowadays, with the rise in average life expectancy, the rate of hospitalization of the older population in intensive care unit (ICU) is gradually increasing. Unfortunately, there are no ideal combination of prognostic factors predicting the mortality in older patients admitted to the ICU. In the present study, we aim to determine the prognostic factors and their impacts on short-time mortality in older critically ill patients.

**Materials and methods:**

This retrospective cohort study was performed between January 2019 and February 2020. We included 133 patients aged ≥80 years and hospitalized ≥24 h in the ICU.

**Results:**

A total of 133 critically ill patients enrolled in the present study. And, the median age of the patients was 85 (80–106) years. 30-days and overall ICU mortality rates were found 30.1% and 34.6%, respectively. The patients were grouped as survivors (n = 94) and nonsurvivors (n = 39). Hospital length of stay before the ICU admission was found significantly longer in nonsurvivors (p = 0.001). Sequential organ failure assessment (SOFA) score and acute physiology and chronic health evaluation-II (APACHE-II) score were significantly higher in nonsurvivors (p < 0.001, p < 0.001). Also, blood lactate level and glucose level were respectively significantly higher in nonsurvivors (p < 0.001, p = 0.006). We found that modified nutrition risk in critically ill (mNUTRIC) score and prehospital clinical frailty scale (CFS) were independent prognostic factors for the older critically ill patients (HR = 9.19, 95% CI=1.47–57.32, p = 0.018, HR = 20.16, 95% CI = 2.63–54.07, p =0.004).

**Conclusion:**

mNUTRIC score and prehospital CFS score were the most important prognostic factors in the admission of older patients to intensive care units.

## 1. Introduction

As a result of decrease in mortality and childbirth rates in the worldwide, the world population getting older. With the rising life expectancy, the number of older patients admitted to medical services has been rising in countries with a growing population due to the increased prevalence of chronic morbidity and functional impairment. Consequently, the admission rates of the older patients to the intensive care unit (ICU) have increased [1–5].

Currently, there are no ideal combination of prognostic factors predicting the mortality in older patients admitted to the ICU. Although, it has been thought that mortality increases in parallel with the age of patients who are admitted to the ICU, recent clinical studies also shown that age is not a crucial prognostic factor for mortality in older patients [3,4,6].

In addition, traditional disease severity scoring systems may not be able to predict the mortality in older patients admitted to the ICU. Because, these scoring systems may not provide significant data about a patient’s prehospital clinical status, such as cognitive impairment, decreased functional capacity, and frailty. These prehospital data may be important markers of mortality and morbidity, especially in the older patients admitted to the ICU. For this reason, there is a need for specific scoring systems that can be used in older patients instead of traditional scoring systems that improve better outcomes [6–8].

Considering the above points, in the present study, we aimed to evaluate the clinical characteristics and outcomes of the older critically ill patients, and also determine the prognostic factors and their impacts on 30-days overall mortality.

## 2. Materials and methods

### 2.1. Study design

**Figure 1:**
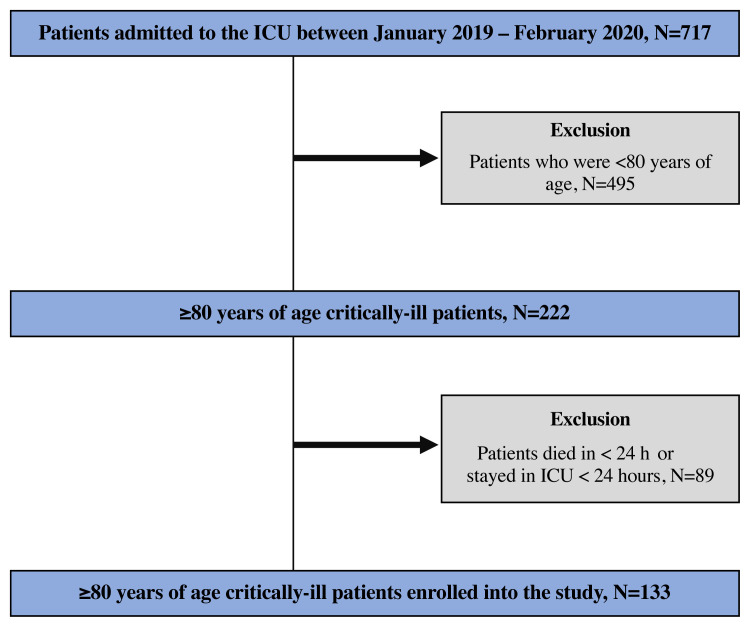
Flowchart of the study.

This single-center retrospective cohort study was performed in a 12-beds adult tertiary ICU of Malatya Training and Research Hospital between January 2019 and February 2020. A total of 717 patients were admitted to the ICU during the study period. A total of 222 patients aged 80 years and older in the ICU were assessed for eligibility. Of these, 89 were excluded from the study due to various reasons. And a total of 133 patients were enrolled in the study (Figure 1).

### 2.2. Data collection and definitions

The following data were recorded and analyzed: all patients’ demographic and clinical data, laboratory findings, types of respiratory support, the reason for admission, admission source, treatment options, hospital length of stay (LOS) before ICU admission, ICU length of stay (LOS), outcomes, scores on the acute physiology and chronic health evaluation-II (APACHE-II) and sequential organ failure assessment (SOFA), modified nutrition risk in critically ill (mNUTRIC) and clinical frailty scale (CFS). Patients’ data and scores reported in this study were collected within the first 24 h following the ICU admission.

The nutritional status of the patients was assessed according to the mNUTRIC score within the first 24 h of the ICU admission. mNUTRIC score includes the following variables: age, number of comorbidities, days from hospital to ICU admission, APACHE-II score, and SOFA score. And the patients with mNUTRIC scores ≥5 were defined as high nutritional risk [9].

The prehospital frailty status of the patients was assessed according to the CFS [10]. And the patients who had CFS ≥5 were defined as frail. CFS was calculated based on patients’ medical records and the interview of the patients and/or their relatives.

APACHE-II and SOFA scores were used for the assessment of the severity of illness. These scores were calculated based on the patients’ worst clinical and laboratory findings observed during the first 24 h following the admission of ICU [11,12]. And also, patients’ Glasgow coma scale (GCS) was calculated at the time to the admission of ICU.

### 2.3. Measurement of outcome

All of the patients were followed during their ICU stay or until death. All-cause of short time mortality was defined as death within 30 days after the ICU admission. Patients’ mortality data were collected from the hospital medical record system.

### 2.4. Statistical analysis

SPSS for Windows 22.0 software (SPSS Inc., Chicago, IL, USA) was used for the statistical analysis of the data obtained from the hospital database. All results were analyzed with a confidence interval level of 95% and a significance level of p < 0.05. The homogeneity and distribution of the variables were assessed using the Skewness–Kurtosis. Frequencies and percentages were used for the categorical data, continuous variables were presented as mean +/− standard deviation or median (min-max) according to the distribution normality of data. We compared the variables between survivors and nonsurvivors. Categorical variables were compared using the chi-squared test. The independent samples t-test was used for the analysis of the two independent groups parametric data while Mann–Whitney U test was used for the analysis of nonparametric data. We used the receiver operating characteristic (ROC) curve to estimate the appropriate cut-off values of SOFA, APACHE-II, CFS, and mNUTRIC score for determining the 30-days mortality. The Kaplan–Meier method was used to determine the overall survival rates of the patients at day 30 and the long-rank test was used to compare the differences in survival between the groups. After the univariate survival analysis, Cox regression analysis was used for the assessment of the multivariate survival analysis.

## 3. Results

### 3.1. Characteristics of the patients

A total of 133 critically ill patients were included in the study. Seventy-five (56.4%) of patients were female and the median age of the patients was 85 (80–106) years. Hypertension (68.4%), coronary artery disease (41.3%), dementia (35.3%), and chronic obstructive pulmonary disease (24.4%) were the most common comorbidities. The emergency department (55.6%) was the most frequent patient’s source of admission to the ICU. Sepsis (32.3%) was the most frequent cause of the admission to the ICU followed by acute cardiogenic pulmonary edema (19.5%) and postoperative respiratory failure (12.8%). Hospital LOS before the ICU admission was found 1.95 ± 4.40 days and ICU LOS was found 11.40 ± 12.76 days.

The patients were grouped as survivors (n = 94) and nonsurvivors (n = 39). We found that there were no statistical differences between the survivors’ and the nonsurvivors’ group with respectively age, sex and diagnosis at ICU admission. However, hospital LOS before the ICU admission was found significantly longer in nonsurvivors (p = 0.001). And also, ICU LOS was found significantly longer in survivors’ group (p = 0.015). We found that SOFA, APACHE-II, CFS and mNUTRIC score were significantly higher in nonsurvivors (p < 0.001). In addition, blood lactate level and glucose level were respectively significantly higher in nonsurvivors (p < 0.001, p = 0.006). Comparison of the baseline clinical characteristics and laboratory findings of the two groups are presented in Tables 1 and 2.

We found that a total of 43 (32.4%) patients underwent invasive mechanical ventilation and 33 (24.8%) patients underwent noninvasive mechanical ventilation. Nonsurvivors had more invasive mechanical ventilation requirements (p < 0.001). Also, the reintubation rate was found higher in nonsurvivors’ group (5.3% vs. 10.2%), but it was not statistically significant (p = 0.3). The use of vasoactive agents was found significantly higher in nonsurvivors (p = 0.004). Treatment options of the patients were presented in Table 3.

### 3.2. Short time survival analysis of the patients

**Figure 2:**
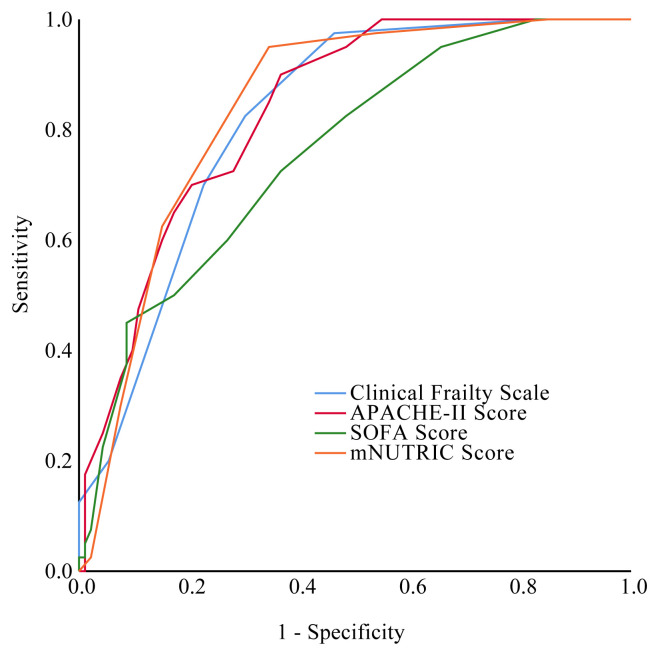
Receiver operating characteristic curves of different scores predicting short time mortality.

We performed ROC curve analysis for finding the optimal cut-off value for determining the 30-days mortality in the older critically ill patients (Figure 2). The cut-off values of SOFA, APACHE-II, CFS, and mNUTRIC scores were presented in Table 4.

In the present study, we found that 30-days and overall ICU mortality rates were found at 30.1% and 34.6%, respectively. Prognostic factors affecting the 30-days mortality in the older patients are presented in Table 5. The effects of the clinical characteristics and laboratory data on the 30-days survival showed that patients with hyperglycemia and hyperlactatemia during the ICU admission had significantly shorter survival times respectively (p = 0.001, p = 0.023). Also, we found significantly shorter survival time in patients with CFS ≥5 (Figure 3A), mNUTRIC score ≥5 (Figure 3B), SOFA score >5 (Figure 3C), and APACHE-II scores ≥23 (Figure 3D) (p < 0.001).

After the univariate survival analysis, we used multivariate Cox regression analysis for determining the independent risk factors of 30-days mortality. It was shown that prehospital CFS (HR = 20.16, 95% CI = 2.63–54.07 p = 0.004) and mNUTRIC score (HR = 9.19, 95% CI = 1.47–57.32, p = 0.018) were independent and significant prognostic factors for the 30-days mortality (Table 6).

## 4. Discussion

In the present study, we evaluated the clinical characteristics and the outcomes of the older critically ill patients. The main finding of our study showed that mNUTRIC score and prehospital CFS score were the most important independent prognostic factors in the admission of the older patients to intensive care units.

Nowadays, the rate of hospitalization of the older population in ICU is gradually increasing with the increase in the average life expectancy [13]. Nielson et al. showed in their study in 2014 that 12.6% of the patients who were admitted to the ICU consisted of patients aged 80 and over, and that patients aged 80 and over who were admitted to intensive care increased by 18% over the years [14]. In our study, 21.1% of the patients who were admitted to the ICU during 13-months aged 80 years and over. The high percentage of ≥80-year-old patients in the present study can be explained by the characteristic of the population in the city.

Problems are experienced in the follow-up of the older patients in ICU due to the consideration that life expectancy will be short as well as due to the underlying comorbidities [13,15]. It has been demonstrated in numerous clinical studies that mortality increases in parallel with the age of patients who are admitted to the ICU, and age is an independent risk factor for mortality [3,16]. However, recent studies have shown that chronological age is not an independent risk factor for mortality. Besides, it has been underscored that rather than chronological age, biological age is more important for survival [6,15,17].

Many studies evaluating the survival in critically ill older patients are single-centered retrospective cohort studies and include different age and disease groups. Therefore, 30-days and ICU mortality rates vary in studies. In previous studies, the mortality rate soars to 50% in critically ill older patients in ICU [1,4,6,15–17]. In the present study, we found that 30-days and overall ICU mortality rates were found 30.1% and 34.6%, respectively.

In the present study, the general ward was the primary admission source for the older patients admitted to the ICU. Also, we found that hospital LOS before the ICU admission was 1.95 ± 4.40 days and ICU LOS was 11.40 ± 12.76 days. Clinical studies that evaluate the association between hospital LOS before the ICU admission and survival of the patients transferred to ICU showed that patients with longer hospital LOS before the ICU admission had worse outcomes and survival. Thus, to improve outcomes in critically ill older patients who have been admitted to ICU, it may be beneficial to establish rapid response teams to rapidly recognize the clinical deterioration of patients who are followed up in general wards, and to make early interventions [18,19].

Sepsis, defined as life-threatening organ dysfunction resulting from the dysregulated response of the host to infection, is one of the most important causes of admission to ICU and mortality. Advanced age is a significant risk factor for the development of sepsis, and a dramatic increase is seen in the incidence of sepsis, particularly in those aged 80 and over [6,15]. In the present study, we found that sepsis (32.3%) was the most frequent cause of admission to the ICU followed by acute cardiogenic pulmonary edema (19.5%) and postoperative respiratory failure (12.8%).

There are no prognostic factors and combinations that can be used during the admission of the older critically ill patients to intensive care units, that are accepted by all clinicians, and whose validity and reliability have been proven in the literature. The fact that the clinical characteristics, which are used in conventional disease severity scorings, such as the APACHE-II score, may be inadequate to accurately predict the survival of critically ill patients, is increasingly gaining acceptance [6,8,20]. We found out that SOFA and APACHE-II scores were not an independent and significant prognostic factor for 30-days survival, albeit we determined that patients with higher SOFA and APACHE-II scores in our study had higher mortality and shorter survival rates. Also, we found that patients with higher serum levels of glucose and lactate had a shorter survival time. However, these two variables were not independent and significant prognostic factors for the 30-days survival.

Traditional disease severity scoring systems may not be able to detect significant data about a patient’s preillness state, such as cognitive impairment, decreased functional capacity, and frailty. However, these data may be important markers of mortality and morbidity, especially in the older patients. Assessing the clinical frailty of patients before admission to the ICU, especially in critically ill patients, could facilitate better clinical decisions [4,6,8].

The concept of clinical frailty is defined as a multivariate syndrome characterized by loss of physical, physiological, and cognitive reserves rather than an acute disease state that increases with age but is not specific to advanced age. Frail elders experience difficulty in adapting to various stressful situations such as acute illness and trauma. Frailty can be assessed using the CFS developed by Rockwood et al. CFS is a 9-point scale and a patient with a score ≥ of 5 is defined as frail [4,8,10,21,22].

The prevalence of prehospital frailty varies in previous studies and it increases with age. More importantly, it has been suggested that an increased CFS score (CFS score of ≥5) is a significant factor in short-term and long-term mortality in the older patients who have been admitted to ICU [3,4,8,21,22]. In the present study, a total of 82 (61.6%) critically ill patients had a CFS score ≥5. Also, we found that the CFS score was significantly higher in nonsurvivors (p < 0.001) and independent prognostic factors of 30-days mortality in the older patients admitted to the ICU included a high CFS score (CFS score ≥5).

The majority of the patients who apply to the ICUs and particularly those who receive mechanical ventilation support are at nutritional risk. In particular, this risk increases even more in fragile and critically ill older patients. Hence, the nutritional risk should be assessed in all patients who have been admitted to the ICU without wasting time [1,6,21]. Based on the mNUTRIC score developed by Heyland et al., patients are divided into low (0–4) and high (5–9) risk groups to evaluate the nutritional risk in critical patients, and early nutritional support is recommended for patients in the high-risk group [9,23]. Moreover, various studies have revealed that a high mNUTRIC score (mNUTRIC score ≥5) is also associated with increased mortality and unfavorable outcomes. For this reason, critically ill patients with high nutritional risk should be identified without delay and early nutritional support should be initiated [24,25]. In the present study, a total of 89 (66.9%) critically ill patients had mNUTRIC score ≥5. Also, we found that patients with mNUTRIC score ≥5 had a shorter survival time.

In conclusion, prehospital CFS and mNUTRIC score were the independent and significant prognostic factors for the older critically ill patients. In addition to traditional scoring systems that assess organ failure during admission of the older patients to the ICU, assessment of prehospital frailty and nutritional risk status could be more effective in predicting short and long-term mortality. Therefore, we recommend that prehospital frailty and the nutritional risk assessment of the older patient should be routinely performed for the more rational use of intensive care unit beds and sufficient prognostic evaluation in these patient groups.

## Figures and Tables

**Figure 3A:**
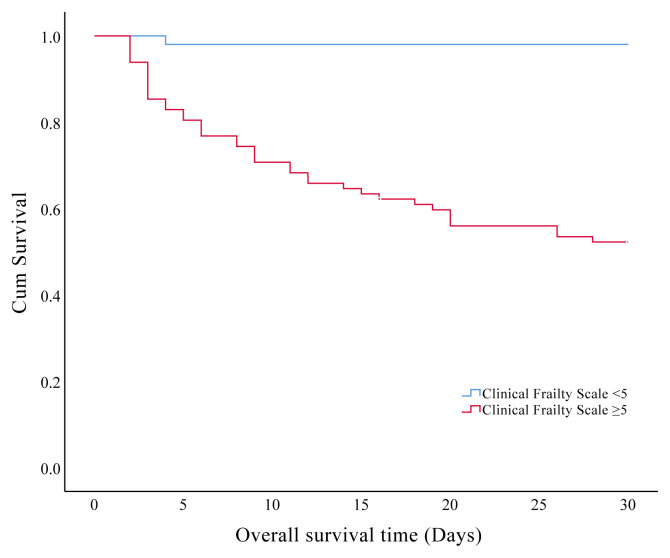
Kaplan–Meier curves of 30-days survival analysis shows the impact of clinical frailty scale in the older critically ill patients.

**Figure 3B:**
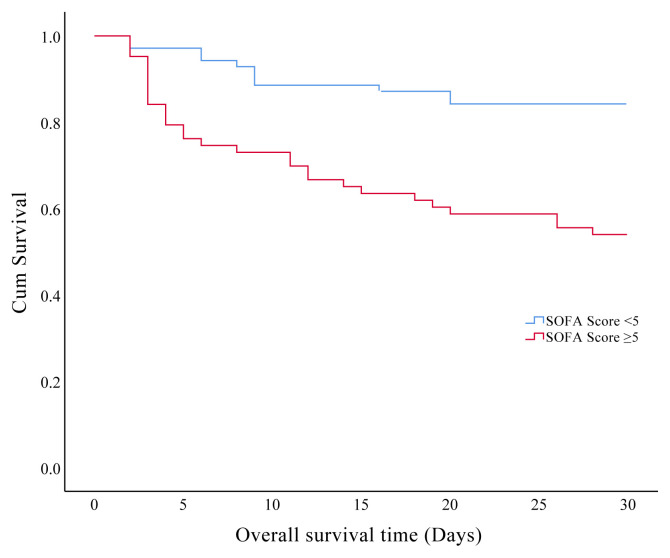
Kaplan–Meier curves of 30-days survival analysis shows the impact of modified nutrition risk in critically ill score in the older critically ill patients.

**Figure 3C:**
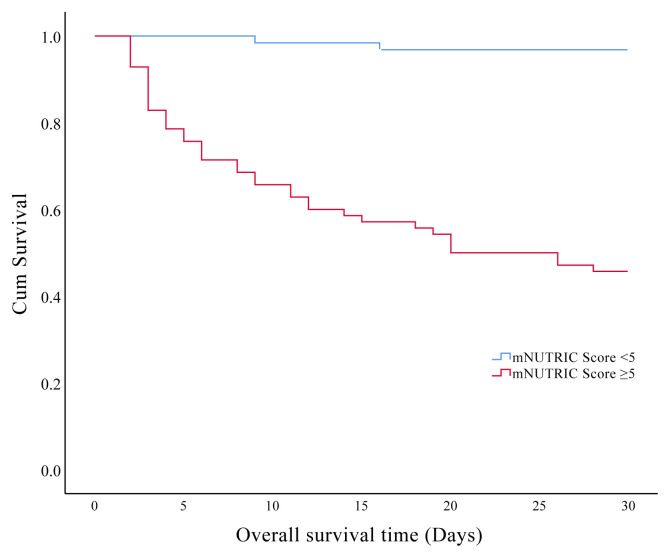
Kaplan–Meier curves of 30-days survival analysis shows the impact of sequential organ failure assessment score in the older critically ill patients.

**Figure 3D:**
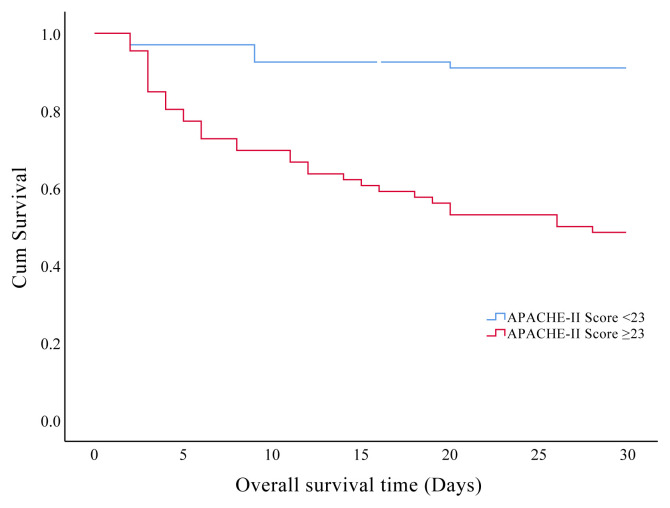
Kaplan–Meier curves of 30-days survival analysis shows the impact of acute physiology and chronic health evaluation-II score in the older critically ill patients.

**Table 1:** Baseline characteristics of the patients.

		All patients (n = 133)	Survivors (n = 94)	Nonsurvivors (n = 39)	*p* value
**Median age, years (min-max)**		85 (80–106)	84.50 (80–106)	86 (80–97)	0.481
**Sex, n (%)**	Male	58 (43.6%)	36 (38.2%)	22 (56.4%)	0.055
	Female	75 (56.4%)	58 (61.8%)	17 (43.6%)	
**Comorbid disease, n (%)**	Hypertension	91 (68.4%)	59 (62.7%)	32 (82.0%)	0.836
	Dementia	47 (35.3%)	35 (37.2%)	12 (30.7%)	0.105
	COPD	37 (27.8%)	21 (22.3%)	16 (41.0%)	0.271
	DM	35 (26.3%)	19 (20.2%)	16 (41.0%)	0.107
	CHF	31 (23.3%)	23 (24.4%)	8 (20.5%)	0.241
	CKD	5 (3.7%)	2 (2.1%)	3 (7.6%)	0.223
	Arrhythmia	23 (17.2%)	17 (18.0%)	6 (15.3%)	0.346
	CVD	20 (15.0%)	14 (14.8%)	6 (15.3%)	0.640
	Malignancy	6 (4.5%)	3 (3.1%)	3 (7.6%)	0.417
	CAD	55 (41.3%)	35 (37.2%)	20 (51.2%)	0.717
**Hospital LOS before ICU admission, days (Me** ± **SD)**		1.95 ± 4.40	1.36 ± 3.51	3.36 ± 5.84	0.001
**ICU LOS, days (Me** ± **SD)**		11.40 ± 12.76	12.36 ± 14.41	9.08 ± 7.05	0.015
**Diagnosis, n (%)**	Sepsis	43 (32.3%)	26 (27.7%)	17 (43.6%)	0.073
	PRF	17 (12.8%)	17 (18.1%)	0	
	ACPE	26 (19.5%)	22 (23.4%)	4 (10.3%)	0.081
	Exacerbation of COPD	9 (6.8%)	8 (8.5%)	1 (2.6%)	0.213
	PTE	3 (2.3%)	1 (1.1%)	2 (5.1%)	0.877
	Cardiac arrest	7 (5.3%)	4 (4.2%)	3 (7.6%)	0.145
	Stroke	15 (11.3%)	7 (7.4%)	8 (20.5%)	0.061
	Miscellaneous	13 (9.7%)	9 (9.6%)	4 (10.3%)	0.040
**Admission source, n (%)**	General ward	36 (27%)	19 (20.3%)	17 (43.5%)	0.005
	Emergency department	74 (55.6%)	53 (56.3%)	21 (53.8%)	0.788
	Operating room	23 (17.4%)	22 (23.4%)	1 (2.7%)	0.003
**Glasgow coma scale, (Me** ± **SD)**		11.54 ± 3.56	12.66 ± 2.89	8.95 ± 3.64	<0.001
**APACHE-II scores, (Me** ± **SD)**		22.77 ± 5.78	20.86 ± 5.18	27.38 ± 4.40	<0.001
**Predicted mortality, % (Me** ± **SD)**		45.64 ± 18.76	39.41 ± 16.55	60.65 ± 14.97	<0.001
**SOFA score, (Me** ± **SD)**		5.83 ± 3.10	5.03 ± 2.78	7.74 ± 3.03	<0.001
**CFS score, (Me** ± **SD)**		5.45 ± 1.70	4.88 ± 1.54	6.82 ± 1.25	<0.001
**mNUTRIC score, (Me** ± **SD)**		5.51 ± 1.69	4.95 ± 1.59	6.87 ± 1.03	<0.001

ICU: intensive care unit; LOS: length of stay; CHF: chronic heart failure; CKD: chronic kidney disease; CVD: cerebrovascular disease; PRF: postoperative respiratory failure; DM: diabetes mellitus; CAD: coronary artery disease; COPD: chronic obstructive pulmonary disease; ACPE: acute cardiogenic pulmonary edema; PTE: pulmonary thromboembolism; APACHE-II: acute physiology and chronic health evaluation II; SOFA: sequential organ failure assessment; CFS: clinical frailty scale; mNUTRIC: modified the nutrition risk in critically ill; Me: mean; SD: standard derivation.

**Table 2:** Baseline laboratory findings of the patients.

	All patients (n = 133)	Survivors (n = 94)	Nonsurvivors (n = 39)	*p* value
**Hb (g/dL)**	11.55 ± 2.80	11.44 ± 2.74	11.81 ± 2.97	0.495
**Ht (%)**	35.96 ± 8.15	35.53 ± 8.33	37.32 ± 7.55	0.384
**Wbc (10** **^3^** **/** **μ** **L)**	12.08 (2.20–303.61)	11.68 (3.04–303.61)	12.85 (2.20–84.80)	0.127
**Lymph (10** **^3^** **/** **μ** **L)**	0.95 (0.14–6.64)	0.96 (0.10–4.33)	0.92 (0.14–6.64)	0.533
**Plt (10** **^3^** **/** **μ** **L)**	230 ± 105	219 ± 89	258 ± 135	0.050
**Urea (mg/dL)**	75 (24–419)	75 (24–419)	76 (36–230)	0.820
**Crea (mg/dL)**	1.32 (0.34–8.20)	1.31 (0.34–8.20)	1.38 (0.74–4.31)	0.328
**AST (U/L)**	29 (6–4384)	29 (6–4384)	27 (14–2142)	0.980
**ALT (U/L)**	18 (4–3062)	19 (4–3062)	15 (5–663)	0.725
**Tot bil (mg/dL)**	0.76 (0.10–3.92)	0.76 (0.10–3.92)	0.72 (0.23–2.62)	0.892
**Glucose (mg/dL)**	150 (69–773)	140 (69–773)	198 (102–510)	0.006
**Albumin (g/dL)**	3.17 ± 0.59	3.22 ± 0.54	3.05 ± 0.68	0.130
**CRP (mg/dL)**	7.19 (0.04–48.00)	7.00 (0.04–48.00)	7.89 (0.09–46.70)	0.127
**pH**	7.34 ± 0.09	7.35 ± 0.09	7.32 ± 0.10	0.183
**Lactate (mmol/L)**	2.10 (0.20–13.80)	1.95 (0.20–9.90)	3.30 (1.20–13.80)	<0.001

CRP: C-reactive protein; AST: aspartate aminotransferase; ALT: alanine aminotransferase; Wbc: white blood cell; Lymph: lymphocytes; Plt: platelets; Hb: hemoglobin; Ht: hematocrit; Tot bil: total bilirubin; Crea: creatinine.

**Table 3:** Treatment options of the patients in the ICU.

		All patients (n = 133)	Survivors (n = 94)	Nonsurvivors (n = 39)	*p* value
**Vasopressor therapy, n (%)**		50 (37.5%)	28 (29.7%)	22 (56.4%)	0.004
**Renal replacement therapy, n (%)**		7 (5.2%)	5 (5.3%)	2 (5.1%)	0.964
**Blood transfusion, n (%)**		72 (54.1%)	55 (58.5%)	17 (43.5%)	0.116
**Respiratory support, n (%)**	**COT**	57 (42.8%)	53 (56.3%)	4 (10.2%)	<0.001
	**NIMV**	33 (24.8%)	24 (24.4%)	9 (23%)	0.765
	**IMV**	43 (32.4%)	17 (19.3%)	26 (66.8%)	<0.001
**Reintubation, n (%)**		9 (6.7%)	5 (5.3%)	4 (10.2%)	0.302
**Tracheostomy, n (%)**		4 (3%)	3 (3.2%)	1 (2.5%)	0.847

ICU: intensive care unit; COT: conventional oxygen therapy; NIMV: noninvasive mechanical ventilation; IMV: invasive mechanical ventilation.

**Table 4:** The optimal cut-off value of clinical frailty scale, mNUTRIC score, SOFA score, APACHE-II scores for the prediction of mortality.

	AUC	95% CI	Sensitivity	Specificity	Cut-off value	p value
**SOFA score**	0.759	0.674–0.845	73.8%	62.8%	5	< 0.001
**APACHE-II scores**	0.835	0.768–0.902	84.6%	64.9%	23	< 0.001
**CFS score**	0.822	0.752–0.892	82.1%	69.1%	5	< 0.001
**mNUTRIC score**	0.841	0.774–0. 908	95.9%	64.9%	5	< 0.001

APACHE-II: acute physiology and chronic health evaluation II; SOFA: sequential organ failure assessment, CFS: clinical frailty scale, mNUTRIC: modified the nutrition risk in critically ill, AUC: area under curve, CI: confidence interval.

**Table 5:** Comparison of mean survival time according to demographic characteristics and ICU parameters.

		Mean survival time (days ± SE)	95% CI		p value
			Lower bound	Upper bound	
**Sex**	Male	22.00 ± 1.44	19.17	24.83	0.074
	Female	25.26 ± 1.08	23.14	27.38	
**Admission source**	General ward	19.88 ± 1.90	16.15	23.61	0.002
	Emergency department	24.15 ± 1.17	21.84	26.46	
	Operating room	29.04 ± 1.17	27.21	30.61	
**Vasoactive agent requirement**	Yes	20.10 ± 1.71	16.74	23.45	0.001
	No	26.10 ± 0.89	24.35	27.84	
**Type of respiratory support**	COT	28.47 ± 0.76	26.97	29.97	<0.001
	NIMV	23.84 ± 1.86	20.19	27.48	
	IMV	17.72 ± 1.71	14.36	21.07	
**Clinical frailty scale**	< 5	29.49 ± 0.50	28.50	30.48	<0.001
	≥ 5	20.31 ± 1.25	17.86	22.79	
**SOFA score**	< 5	26.81 ± 0.92	24.99	28.62	<0.001
	≥ 5	20.55 ± 1.45	17.70	23.41	
**APACHE-II scores**	< 23	28.07 ± 0.78	26.53	29.60	<0.001
	≥ 23	19.56 ± 1.42	16.77	22.34	
**mNUTRIC score**	< 5	29.44 ± 0.39	28.67	30.21	<0.001
	≥ 5	18.81 ± 1.39	16.07	21.55	
**Hyperglycemia**	Yes	20.03 ± 2.25	15.61	24.46	0.001
	No	26.73 ± 0.91	24.94	28.52	
**Hyperlactatemia**	Yes	21.89 ± 1.32	19.29	24.48	0.023
	No	26.30 ± 1.03	24.27	28.32	
**Hypoalbuminaemia**	Yes	22.12 ± 1.58	19.02	25.22	0.077
	No	24.81 ± 1.04	22.76	26.87	

CI: confidence interval, SE: standard error; SOFA: sequential organ failure assessment, APACHE-II: acute physiology assessment and chronic health evaluation II, mNUTRIC: modified the nutrition risk in critically ill, CFS: clinical frailty scale, COT: conventional oxygen therapy; NIMV: noninvasive mechanical ventilation; IMV: invasive mechanical ventilation.

**Table 6:** Multivariate Cox regression analysis of 30 days mortality.

	Hazard ratio	95% CI		p value
		Lower bound	Upper bound	
**SOFA score**	0.30	0.07	1.28	0.106
**APACHE-II scores**	3.05	0.78	11.88	0.106
**CFS score**	20.16	2.63	54.07	0.004
**mNUTRIC score**	9.19	1.47	57.32	0.018
**Vasoactive agent requirement**	1.62	0.60	4.39	0.336
**Hyperglycemia**	0.79	0.30	2.07	0.644
**Hyperlactatemia**	0.34	0.10	1.10	0.074

CI: confidence interval, SOFA: sequential organ failure assessment, APACHE-II: acute physiology assessment and chronic health evaluation II, mNUTRIC: modified the nutrition risk in critically ill, CFS: clinical frailty scale.
